# Absence of the Tks4 Scaffold Protein Induces Epithelial-Mesenchymal Transition-Like Changes in Human Colon Cancer Cells

**DOI:** 10.3390/cells8111343

**Published:** 2019-10-29

**Authors:** Bálint Szeder, Júlia Tárnoki-Zách, Dóra Lakatos, Virág Vas, Gyöngyi Kudlik, Balázs Merő, Kitti Koprivanacz, László Bányai, Lilla Hámori, Gergely Róna, András Czirók, András Füredi, László Buday

**Affiliations:** 1 Institute of Enzymology, Research Centre for Natural Sciences, Hungarian Academy of Sciences, 1117 Budapest, Hungary; szeder.balint@ttk.mta.hu (B.S.); vas.virag@ttk.mta.hu (V.V.); mero.balazs@ttk.mta.hu (B.M.); koprivanacz.kitti@ttk.mta.hu (K.K.); banyai.laszlo@ttk.mta.hu (L.B.); hamori.lilla@ttk.mta.hu (L.H.); andras.fueredi@meduniwien.ac.at (A.F.); 2 Department of Biological Physics, Eötvös University, 1117 Budapest, Hungary; zachjuli@yahoo.fr (J.T.-Z.); burek43@gmail.com (D.L.); aczirok@kumc.edu (A.C.); 3 Department of Biochemistry and Molecular Pharmacology, New York University School of Medicine, New York, NY 10016, USA; gergely.rona@nyumc.org; 4 Department of Anatomy and Cell Biology, University of Kansas Medical Center, Kansas City, KS 66160, USA; 5 University of Kansas Cancer Centre, Kansas City, KS 66160, USA; 6 Institute of Cancer Research, Medical University of Vienna, 1090 Vienna, Austria; 7 Department of Medical Chemistry, Semmelweis University Medical School, 1094 Budapest, Hungary

**Keywords:** Tks4, scaffold protein, EMT, HCT116, motility, invasiveness

## Abstract

Epithelial to mesenchymal transition (EMT) is a multipurpose process involved in wound healing, development, and certain pathological processes, such as metastasis formation. The Tks4 scaffold protein has been implicated in cancer progression; however, its role in oncogenesis is not well defined. In this study, the function of Tks4 was investigated in HCT116 colon cancer cells by knocking the protein out using the CRISPR/Cas9 system. Surprisingly, the absence of Tks4 induced significant changes in cell morphology, motility, adhesion and expression, and localization of E-cadherin, which are all considered as hallmarks of EMT. In agreement with these findings, the marked appearance of fibronectin, a marker of the mesenchymal phenotype, was also observed in Tks4-KO cells. Analysis of the expression of well-known EMT transcription factors revealed that Snail2 was strongly overexpressed in cells lacking Tks4. Tks4-KO cells showed increased motility and decreased cell–cell attachment. Collagen matrix invasion assays demonstrated the abundance of invasive solitary cells. Finally, the reintroduction of Tks4 protein in the Tks4-KO cells restored the expression levels of relevant key transcription factors, suggesting that the Tks4 scaffold protein has a specific and novel role in EMT regulation and cancer progression.

## 1. Introduction

Cellular plasticity enables cells to undergo major phenotypic changes under certain physiological conditions. One such process is epithelial-mesenchymal transition (EMT). EMT occurs in multiple biological processes, including wound healing, organ formation, embryogenesis, and the formation of solid metastases. This transition is not necessarily permanent as the mesenchymal characteristics can be reverted back to an epithelial phenotype [1,2,3,4,5].

Epithelial cells undergoing EMT lose their apico-basal polarity and their cobblestone-like arrangement disappears to give rise to an elongated spearhead-like morphology [6]. The expression of cell–cell junction proteins is repressed, while the synthesis of extracellular matrix proteins (ECM) is initiated [7]. This proteome remodeling is mainly facilitated by E-box-binding transcription factors, such as Snail1 and Snail2/Slug, which downregulate E-cadherin expression [8,9,10]. Furthermore, Snail1/2 and Zeb1/2 can repress other tight junction components, such as connexins, occludins, and ZO-1, among others [11]. The Twist family also plays a part in regulating E-cadherin expression by promoting the expression of Snail transcription factors [12]. Simultaneously increased expression of mesenchymal markers, including vimentin, fibronectin, and N-cadherin, leads to complete transformation [13]. Many signaling pathways can lead to EMT. Traditionally, developmental pathways have been viewed as key players in this signal initiation, e.g., TGF-β, WNT, and NOTCH signaling, as well as pathways involving various receptor tyrosine kinases (RTK) [14,15,16,17,18,19,20], including EGF, which can also facilitate EMT induction [14,20].

Scaffold proteins control cellular signaling by interacting with and bringing various pathway components, such as enzymes and regulatory proteins, into close proximity [21,22]. The protein product of the *SH3PXD2B* gene, Tks4, belongs to the family of scaffold proteins [23]. Tks4 is involved in podosome formation, cell migration, mesenchymal stem cell differentiation, adipose tissue beigeing, and bone trabecular formation [23,24,25,26,27,28,29]. Inactivating mutations in the *SH3PXD2B* gene cause a rare genetic disorder known as Frank-ter-Haar syndrome (FTHS, OMIM:249420) [30]. FTHS patients show several serious symptoms related to altered tissue development, such as cardiac deficiencies, kyphosis, shortened and bowing long bones, and craniofacial and dental abnormalities [31,32,33,34,35].

Tks5, a homolog of Tks4, has been implicated in cancer progression [36]. Matrigel invasion assays with various human cancer cells revealed that Tks5 expression is vital for invadopodium formation [36]. Further studies have demonstrated the clinical significance of Tks5 in a number of different cancer types, including breast cancer, gliomas, and lung adenocarcinoma, as well as colon and prostate cancer [37,38,39,40]. An elegant series of recent experiments showed that both Tks family members (Tks4 and Tks5) play key roles in melanoma cell invasion and metastasis [27]. Furthermore, both Tks proteins are highly expressed in human melanoma tissue, suggesting that the Tks proteins are important regulators of melanoma growth [27].

In our study, the role of Tks4 in colon cancer cells was investigated. The scaffold protein was deleted via the CRISPR/Cas9 system, and the effects of Tks4 deletion were investigated via a number of different methods, including the characterization of cell morphology and motility, cell adhesion, and spheroid formation, as well as the measurement of the expression levels of EMT-governing master transcription factors. Our results show that loss of Tks4 in colon cancer cells induces an EMT-like mesenchymal phenotype.

## 2. Materials and Methods

### 2.1. CRISPR/Cas9-Mediated Engineering of the HCT116 Cell Genome

HCT116 cells were maintained in McCoy’s 5A medium (Gibco, Paisley, UK) supplemented with 10% fetal bovine serum (FBS; Gibco) and antibiotics, penicillin and streptomycin (Sigma-Aldrich, Schnelldorf, Germany). Cell number and viability were determined by the TC20 Automated Cell Counter (Bio-Rad, Hercules, CA, USA) using 0.4% trypan blue dye exclusion. Cells were tested routinely for Mycoplasma infection (MycoAlert™ mycoplasma detection kit, Lonza). Morphological assessment was performed using an Olympus CKX41 inverted microscope.

HCT116 cells were transfected with pCMV-Cas9-GFP_SH3PXD2B (Sigma-Aldrich) using FuGENE HD (Promega, Madison, WI, USA) transfection reagent. Two days after transfection, cells were passaged and sorted for GFP expression (Attune FACSARIA III sorter). After sorting, the GFP-positive cells were seeded as single-cell colonies (1 cell/100 μL) into three 96-well plates. After reaching confluency, cells were expanded and subjected to genotyping where genomic DNA (gDNA) was isolated using the MasterPure DNA Purificaton Kit (Epicentre) following the manufacturer’s instructions.

DNA fragments of various sizes that covered the gRNA target region were amplified using the primers: E2P2_F: ATAAGAATTCATTGTTTTCTGTGCGTGCCG and E2P2_R: TATGGATCCGCTCACCAGCAAACACGATT. The PCR products were purified and digested with Eco72I (Thermo Scientific), which has a digestion site that incorporates two nucleotides from the PAM sequence and is, therefore, disrupted if Cas9 cleavage takes place (Figure S1). To confirm that the colonies had mutations in both alleles, PCR products, that Eco72I was unable to digest, were sub-cloned into the pBluescript II SK(+) plasmids and amplified in a bacterial host. Plasmid DNA was isolated from individual colonies and then sequenced.

### 2.2. Cell Adhesion Assays

Vybrant Cell Adhesion Assays Kit (Molecular Probes) was used for cell adhesion measurements. Cells were trypsinized, washed twice with phosphate-buffered saline (PBS), and then resuspended in serum-free McCoy’s 5A medium (Gibco). The cells were labeled with calcein-AM dye (Sigma-Aldrich) at a concentration of 0.25 μM for 30 min at 37 °C and then seeded into 96-well plates at a density of 10,000 cells/well. The plates were incubated for three hours, after which the non-adherent labeled cells were carefully washed away with 200 μL of pre-warmed McCoy’s 5A medium. This washing step was repeated three times. Finally, the medium was decanted and the wells were filled with 200 μL of PBS. The fluorescence was measured at 517 nm using empty PBS-filled wells as a negative control.

### 2.3. Antibodies

Antibodies against Tks4 were purchased from Merck-Millipore (09-267) and Sigma-Aldrich (A303-437A), the generation of the polyclonal anti-Tks4 antibody was described earlier [41]. Antibodies α-tubulin (DM1A) and fibronectin (F7387) were obtained from Sigma-Aldrich. Antibodies against E-cadherin (ab11512 and ab 15148), fibronectin (ab45688), and Snail1 (ab216347) were purchased from Abcam.

### 2.4. Confocal Microscopy

Cells were seeded into 8-well μ-Slides (ibidi) at a density of 20,000 cells/well. The next day, the medium was decanted and the cells were washed three times with 200 μL of pre-warmed PBS. The cells were fixed in 4% para-formaldehyde solution for 20 min at room temperature, washed, and then blocked with complete blocking solution (0.5% BSA, 5% FBS, 0.1% TritonX-100 in sterile PBS) for one hour at room temperature. They were then incubated with the relevant primary antibodies for one hour. After incubation, the cells were washed with PBS, and the corresponding secondary antibodies were added, followed by incubation for one hour at room temperature. Imaging was carried out using a ZEISS LSM-710 system (Carl Zeiss microscopy Gmbh, Jena, Germany) with a 40×/1.4 Plan-Apochromat oil immersion objective. Images were processed with ZEN (Carl Zeiss microscopy Gmbh, Jena, Germany).

### 2.5. Extraction of Total RNA and RT-PCR Analysis

WT and Tks4-KO cells were lysed using TRIzol™ Reagent (Life Technologies, Carlsbad, CA, USA). The total RNA was isolated using the Direct-zol^®^ MiniPrep kit (Zymo Research, Irvine, CA, USA), which was also used to prepare the total RNA samples. gDNA contamination was prevented via in-column DNAse treatment. Complementary DNA (cDNA) was produced via reverse transcription of 1 μg of RNA using the First Strand cDNA Synthesis kit for RT-PCR (Roche, Basel, Switzerland) with the provided random primers. The real-time PCR analyses were done using the StepOne™ Real-Time PCR System (Life Technologies). Fold changes were determined via the 2^−ΔΔCt^ method. Relative expression levels are presented as the mean values ± S.D. of three independent experiments. Pre-developed TaqMan^®^ assays for FN1 (Hs01549976_m1), SNAI1 (Hs00195591_m1), SNAI2 (Hs00161904_m1), TWIST (Hs01675818_s1) and ACTB (Hs01060665_g1) were purchased from Applied Biosystems.

### 2.6. Western Blot Analysis

Before lysation with ice-cold harvest buffer (30  mM Tris (pH 7.5), with 100  mM NaCl, 1% Triton X-100, 10  mM NaF, 1  mM Na_3_VO_4_, 1  mM EGTA, 2 mM 4-nitrophenyl phosphate, 10  mM benzamidine, 1 mM phenylmethylsulphonyl fluoride, 25  μg/mL Pepstatin A, 25  μg/mL trypsin inhibitor, and 25  μg/mL aprotinin), cells were washed with phosphate-buffered saline solution. Lysates were centrifuged at 14,000 rpm, 10 min at 4 °C. Sample loading was then added to the supernatants, followed by incubation at 95 °C for 10 min. The total protein concentrations were determined using Bradford reagent. 30 µg of each protein sample were subjected to SDS-PAGE using 10% gels. Nitrocellulose membranes were blocked and incubated for 60 min with anti-Tks4 antibody at room temperature. After several washing steps, the membranes were incubated for 30 min with horseradish peroxidase-conjugated secondary antibody (GE Healthcare) and then washed three times for 10 min each. The proteins of interest were visualized via enhanced chemiluminescence (ECL) detection reagents (Amersham, Little Chalfont, UK). Chemiluminescent imaging was performed with a ChemiDoc MP system (Bio-Rad).

### 2.7. Construction of the pCAGIG_hTks4 Rescue Plasmid and Transient Tks4 Expression

The coding sequence of human Tks4 (hTks4) (Assembly: GCF_000001405.33, locus: NC_000005.10) was sub-cloned into pcDNA3.1/TOPO-V5-His, as described earlier [41]. This construct was used as a template to amplify the Tks4 coding sequence with the primers ATTAGATATCCGTAGAATCGAGACCGAGG and ATTAGAATTCAGCGCCACCATGCCGCCG. The produced amplicon was sub-cloned into pCAGIG (AddGene plasmid id.: #11159) using EcoRI (Thermo Fisher) and BamHI (Thermo Fisher). An internal EcoRI site in the Tks4 coding sequence was mutated from GAATTC to GAGTTC using the Q5 site-directed mutagenesis kit (NEB) according to the manufacturer’s instructions. 100 µL of OptiMem (Gibco) medium was mixed with 3 µL of X-tremeGene transfection reagent (Roche) and 1.6 µg of pCAGIG_hTks4 plasmid. The mixture was incubated at room temperature for 15 min and then added dropwise onto Tks4-KO cells. After 16 h, fresh media was added, and the cells were incubated for an additional 24 h. The next day, the cells were washed with pre-warmed sterile PBS (Sigma-Aldrich), followed by trypsinization and flow cytometry. 100,000 WT and 100,000 Tks4-KO GFP-positive cells were collected and total RNA was isolated using the method described above.

### 2.8. Automated Microscopy

Recording of cellular behavior was performed in a time-lapse manner. Frames were collected via a Leica DM IRB inverted microscope equipped with a motorized stage (Marzhauser SCAN-IM) and a 10× HC-PLAN objective (NA 0.25, working d: 11.0 mm). Frames were digitalized via an Olympus DP70 CCD camera. For the entire duration of the recordings, the cells were maintained at 37 °C in a 5% CO_2_ atmosphere. Phase-contrast images from each microscopic field of view were collected every 10 min for durations up to 5 days post-seeding.

### 2.9. Segmentation of Cell Covered Area

The cell covered areas were identified by the presence of large local variability in brightness values following a previously described method [42,43]. The segmentation and confluency calculating script can be accessed at http://github.com/aczirok/cellconfluency. The cell clusters were detected and cluster size distribution histograms were calculated by python scripts, available at github upon acceptance of the manuscript.

### 2.10. Optical Flow Analysis

Cell movements were calculated by optical flow analysis of consecutive image pairs (particle image velocimetry, PIV) using the method described in [44,45].

### 2.11. Individual Cell Movement

Cells were tracked by a custom software, GTrack [46]. 20 WT and 20 Tks4-KO cells were followed for 25 h in a timeframe corresponding to 60–85 h post-seeding. The motion of individual cells was tracked following a previously described method [47,48].

### 2.12. Spheroid Formation

The spheroids were formed in agarose micromolds. Briefly, 2% boiling agarose solution (Invitrogen) was poured into a PDMS template (3D Petri Dish, Microtissues). The agarose was then allowed to gelate at −20 °C for 5 min. The resulting non-adherent agarose micromolds, each of which contained 35 wells with a diameter and depth of 800 µm, were placed in 35-mm culture dishes and equilibrated in McCoy’s 5A cell culture medium for 1 h. Approximately 3000 cells in suspension were transferred into each well, and then the wells were filled up with cell culture medium. The aggregation process was followed via automated microscopy. Phase-contrast images of aggregates in each microscopic field of view were collected every hour.

### 2.13. 3D Collagen Invasion Assay

Spheroids from WT and Tks4-KO cells were embedded in 1.7 mg/mL type I collagen (Corning) gels, prepared following the manufacturer’s instructions. The gels were kept in 6 mm diameter poly-lactic acid (PLA) wells, 3D printed on 35-mm culture dishes [49]. First, 30 µL collagen I solution was poured into the wells and allowed to form a gel 0.5 mm thick at the center of the well. Then to each well, a few spheroids were added within an additional 30 µL collagen I solution, which was poured on the top of the first collagen I gel layers. After gelation 1 mm thick spheroid-containing collagen I sandwiches were submerged in McCoy’s 5A culture medium and imaging was carried out as described above.

### 2.14. Statistical Analyses

Statistical analyses were performed using Student’s unpaired t-test following testing of the data via the F-test to confirm normal distributions.

## 3. Results

### 3.1. Generation of Tks4 Knockout HCT116 Cells

**Figure 1:**
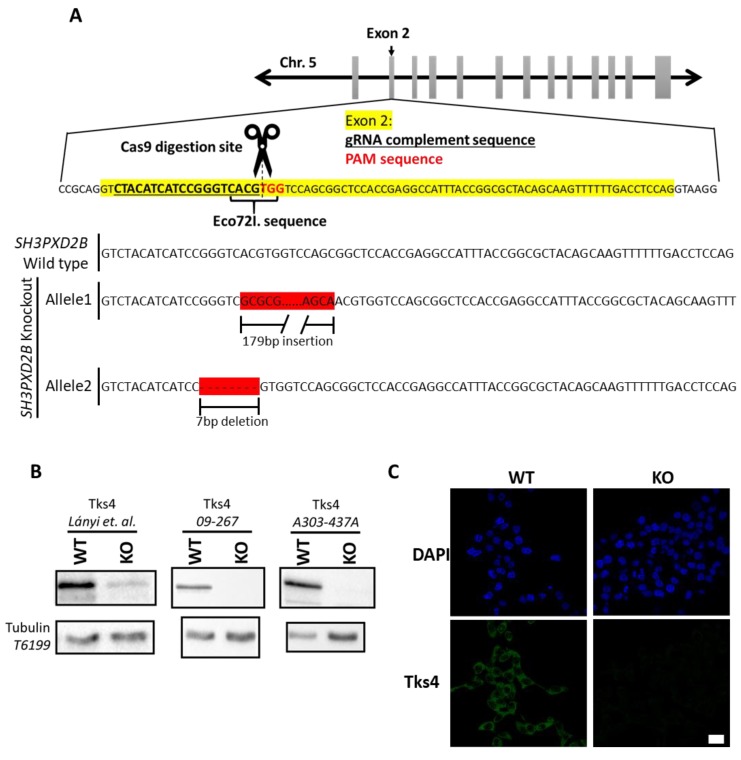
Deletion of *SH3PXD2B* via CRISPR/Cas9 in HCT116 cells. (**A**) Human *SH3PXD2B* is located on chromosome 5. Exon 2 is highlighted with a yellow background, the gRNA complement sequence is underlined, and the PAM sequence is indicated with red nucleotides. *SH3PXD2B* knockout “Allele1” contains a 17-bp long insertion, and “Allele2” contains a 7-bp deletion. (**B**) Validation of the absence of Tks4 protein via Western blotting, with multiple antibodies. (**C**) Representative fluorescent images of wild type and Tks4-KO cell cultures. Nuclei were visualized with DAPI (blue), while Tks4 is shown in green. Scale bar represents 10 µm.

Since the Tks scaffold proteins are involved in cellular migration, invasion, and the progression of several cancer types, including colon cancer, we investigated the role of Tks4 in human colon cancer HCT116 cells by knocking out its gene using the CRISPR/Cas9 system. The genetic alterations were confirmed via sequencing of the affected genomic regions (Figure 1A). The modified exonal sequences were inserted into the wild type open reading frame. Both modified alleles introduce a premature stop codon, thus marking the transcripts for nonsense-mediated decay (Figure S2). The absence of Tks4 in the cells was confirmed via Western blot analysis and immunocytochemistry. The Western blot experiments showed that the Tks4-specific band present in the wild type cell lysate was nearly absent in the Tks4-KO cell clone (Figure 1B).

In the first Western blot image shown in Figure 1B, a faint band in the position of Tks4 remains in the Tks4-KO cell lysate. It is likely that the anti-Tks4 polyclonal antibody recognizes another protein epitope on the gel with the same molecular weight as that of Tks4. To fully exclude the possibility that the Tks4 knockout was not complete, we tested multiple antibodies and immunocytochemistry was also performed (Figure 1B and Figure S3). DAPI staining was used to visualize the cell nuclei, and Tks4 was labeled with the anti-Tks4 antibody. Figure 1C shows that in WT cells, the Tks4 signal is located abundantly in the cytosol, while it is completely absent in the Tks4-KO cells.

### 3.2. Tks4-KO Cells Show Morphological Changes and Increased Expression of Fibronectin and Snail2 Transcription Factor

**Figure 2:**
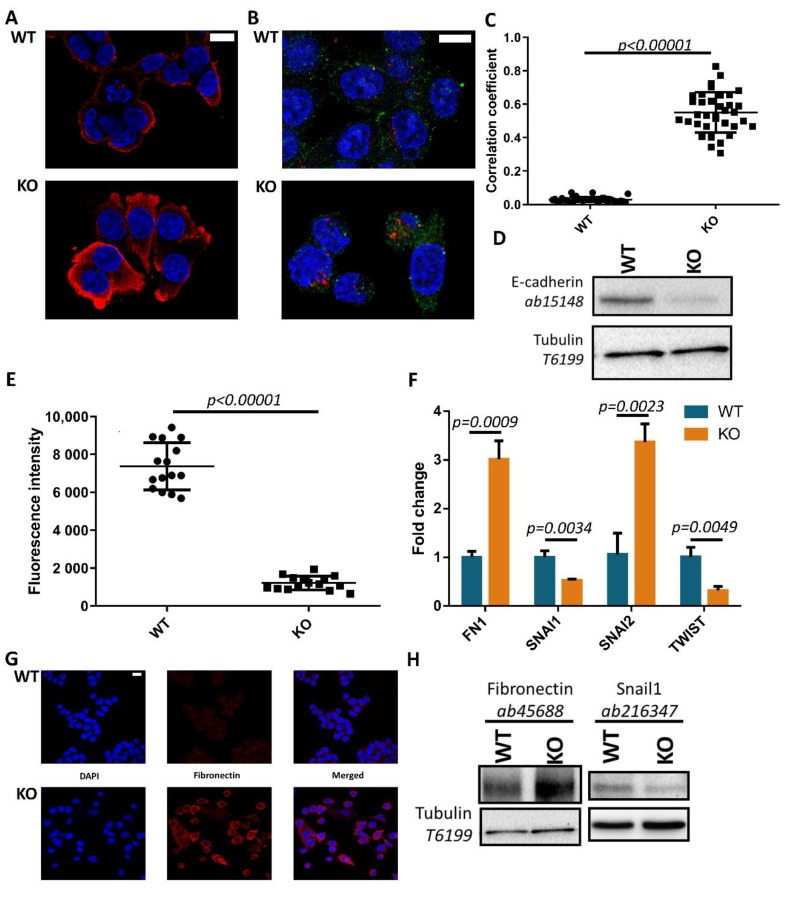
Loss of Tks4 induces morphological changes and altered expression levels of epithelial to mesenchymal transition (EMT) markers. (**A**) E-cadherin (red) staining of wild type and Tks4-KO cells. The nuclei were visualized with DAPI (blue), and the scale bars represent 20 µm (**B**) Staining of E-cadherin (green) and acidic lysosomes (red, LysoTracker™ Dnd-99). The nuclei were stained with DAPI, and the scale bars represent 20 µm. (**C**) Co-localization analysis of the E-cadherin and the lysosomal signals. The center lines show the medians, and the end lines indicate the 25th and 75th percentiles, *n* = 38. Student’s unpaired t-test was used for the statistical analysis. (**D**) Investigation of the E-cadherin protein levels via Western blotting. (**E**) Cellular adherence measurements. Center lines show the medians and the end lines indicate the 25th and 75th percentiles, *n* = 15. Student’s unpaired t-test was used for the statistical analysis. (**F**) Quantitative PCR analyses of the relative expression levels of the EMT-governing transcription factors Snail1 (SNAI1), Snail2 (SNAI2), and Twist (TWIST), and the mesenchymal marker protein fibronectin (FN1). The error bars show the standard deviation, and Student’s unpaired t-test was used for the statistical analysis. (**G**) Fibronectin (red) staining of wild type and Tks4-KO HCT116 cells. The nuclei were stained with DAPI (blue), and the scale bar represents 20 µm. (**H**) Investigation of the E-cadherin protein levels via Western blotting.

The initial examination of morphology in Tks4-KO cells showed elongated soma and decreased circularity compared to the WT cultures (Figure 2A), changes strongly associated with the epithelial-to-mesenchymal transition (EMT) in epithelial cells. Therefore, we investigated the expression and localization of E-cadherin in WT and TKs4-KO cells. Figure 2A shows that both wild type and Tks4-KO cells express E-cadherin, however, the expression pattern is significantly different. WT cells grow in colonies; the cell–cell junctions were tight and syncytium-like structures were formed where the inner boundaries were barely visible. However, in the KO colonies, the cell–cell contacts were more prominent, and individual cells could be easily identified. Furthermore, E-cadherin appeared to be translocated to the cytosol, suggesting that E-cadherin internalization was triggered in Tks4-KO cells (Figure 2A).

Palacios et al. demonstrated that, upon EMT, E-cadherin is internalized by lysosomes and not recycled to the membrane. Depletion of the E-cadherin protein level ensures a lasting disruption of cell−cell contacts and leads to a more motile phenotype [50]. Figure 2B,C demonstrates that in Tks4-KO cells, the E-cadherin signal partly co-localizes with lysosomes. Analyzing the data with Pearson’s correlation showed a correlation coefficient of 0.551 ± 0.120 in the Tks4-KO compared to 0.029 ± 0.014 in the WT. This finding suggests that E-cadherin is partially internalized and found in acidic lysosomes. Western blotting with a second antibody revealed that E-cadherin is indeed downregulated in Tks4-KO cells on the protein level (Figure 2D and Figure S4), thus supporting increased motility in these cancer cells.

The conversion to the mesenchymal phenotype also involves a decrease in cell polarity and increased detachment from the basal membrane [1,51,52]. Therefore, the effect of the absence of Tks4 on the cell-surface adhesion was investigated. Measuring fluorescence intensity of calcein labeled cells, we found that the surface adhesion abilities of Tks4-KO were markedly reduced (Figure 2E).

To assess the changes in the EMT program in the Tks4-KO cells at the mRNA level, qPCR experiments were conducted to measure the expression levels of the Snail1, Snail2, and Twist1 (henceforth referred to as Twist) transcription factors, and fibronectin (FN1), an ECM protein that is an early hallmark of the EMT process. Figure 2F demonstrates that both fibronectin and Snail2 were upregulated in the Tks4-KO cells. In contrast, we noticed that the levels of Snail1 and Twist were slightly, but significantly downregulated in Tks4-KO cells compared to WT. These findings suggest that loss of Tks4 in cancer cells can influence the expression levels of EMT-inducing transcription factors and proteins. To ensure that the expression level changes observed in the qPCR experiments also occurred at the protein level, immunocytochemistry and Western blotting were used. As seen in Figure 2G, wild type HCT116 cells showed low fibronectin expression. In contrast, Tks4-KO cells showed noticeable levels of fibronectin. Figure 2H also shows fibronectin upregulation in the KO cells, while Snail1 downregulation can also be observed (Figure 2H and Figure S4). Taken together, these findings indicate the initiation of a transition towards a more mesenchymal phenotype.

### 3.3. Tks4-KO Cells Showed Increased Motility and Detachment from Each Other in Monolayer Cultures

**Figure 3:**
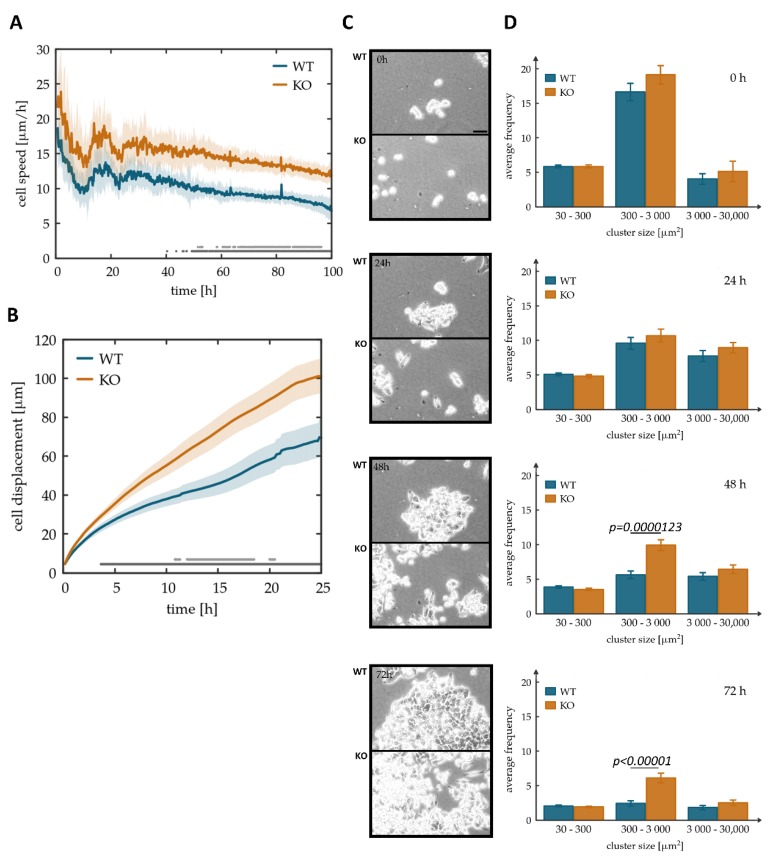
Tks4-KO cells display increased motility and less stable cell-cell adhesions. (**A**) Speed of two-dimensional random motility of WT and Tks4-KO (KO) cells in monolayer cultures, extracted by the optical flow (PIV) method. Solid lines indicate an average of 27 microscopic fields, shaded areas represent the SEM. The reported behavior was observed in *n* = 3 independent experiments. Statistically significant differences between wild type and Tks4-KO cells are indicated at the bottom of the chart. Dark and light gray dots represent a p-value lower than 0.05 and 0.01, respectively, obtained by unpaired Student’s t-tests performed at each time point. (**B**) Net cell displacements during time intervals of various durations. Twenty wild type and Tks4-KO cells were followed manually over a period of 25 h post-seeding. Solid lines indicate the average while the shaded areas represent the SEM. The difference between wild type and Tks4-KO cells is statistically significant for displacements larger than 20 µm. Dark and light gray dots indicate *p*-values lower than 0.05 and 0.01, respectively, established by unpaired Student’s t-tests performed at each time point. (**C**) Representative micrographs of cell clusters, recorded at 0, 24, 48, 72 h post-theseeding. Scale bar represents 40 µm. (**D**) Frequency of cell clusters categorized according to their area. Bar graphs display the average of three independent measurements, statistical analysis was carried out by using unpaired Student’s t-test, error bars show SEM.

To test the functional role of Tks4 in HCT116 cells, we recorded monolayer cultures grown on tissue culture plastic with an automated microscopy system. To investigate if Tks4-KO HCT116 cells exhibit a mesenchymal phenotype with an increased motility, the speed of random two-dimensional motility was estimated by calculating the optical flow with an automatic PIV analysis [44,45]. To avoid the effects of crowding and confluence-induced senescence, data analysis was restricted to the first 100 h post-seeding. The PIV analysis indicated a statistically significant, ~30% increase in motility in the case of Tks4-KO cells (Figure 3A and Figure S5A). This difference in motile activity was present from seeding throughout the 100 h long observation period. As a complementary characterization to the automatic PIV method, we manually tracked the trajectories of 20 WT and Tks4-KO cells using the same image sequence. Cell trajectories were then statistically characterized by calculating their mean net displacement for time windows of various durations (Figure 3B and Figure S5B). This measure of motility also indicated a ~30% increase for Tks4-KO cells for displacements larger than 20 µm; displacements larger than a cell and hence detected reliably by the human operator.

We have shown earlier that in Tks4-KO cells the downregulation of E-cadherin has been initiated, leading to a more mesenchymal morphology (Figure 2B,C). Accordingly, we evaluated the ability of cells to form and remain in clusters, an important function of cadherins. Image sequences that were recorded from WT and Tks4-KO cultures indicated the abundance of single cells and small clusters in the Tks4-KO cultures, while wild type cells formed large clusters (Figure 3C). To quantify the difference, we developed image processing tools to identify each cell cluster within the images. Our analysis indicates that the overall proliferation rate and hence the increase in the cell-covered area is not affected by the absence of Tks4 (Figure S6). Accordingly, cluster size distributions indicate an overall increase in cluster size with time (Figure S7). This measure also indicates a subpopulation of Tks4-KO cells that remain in small clusters. Cluster size distributions, pooled from parallel experiments, indeed indicate an increase in the number of small clusters in Tks4-KO cultures (Figure S7). Overall, we identify a two-fold increase in the frequency of clusters that fall within the 300–3000 µm^2^ range and thus likely contain 1–10 cells (Figure 3D). This preponderance of smaller clusters can reflect increased detachment, motility and decreased cell-cell adhesion. These findings, together with the partial internalization of E-cadherin (Figure 2B,C) suggest that cell-cell connections are impaired in the absence of Tks4.

### 3.4. Tks4-KO Cells Show Decreased Spheroid Forming Potential and Display Increased Invasiveness in Collagen Matrix

Metastasis formation requires cells to detach from the primary tumor, invade the microenvironment and eventually enter and leave the circulation through the processes of intra- and extravasation. The initial step of this process is governed by EMT, where one or multiple cancer cells from the primary solid tumor undergo transition to acquire the motile and less restrained invasive mesenchymal phenotype [4]. To investigate if Tks4-KO cells are indeed more invasive, spheroids were established from both wild type and Tks4-KO cells and subsequently embedded into a 1 mm thick collagen-I gel.

**Figure 4:**
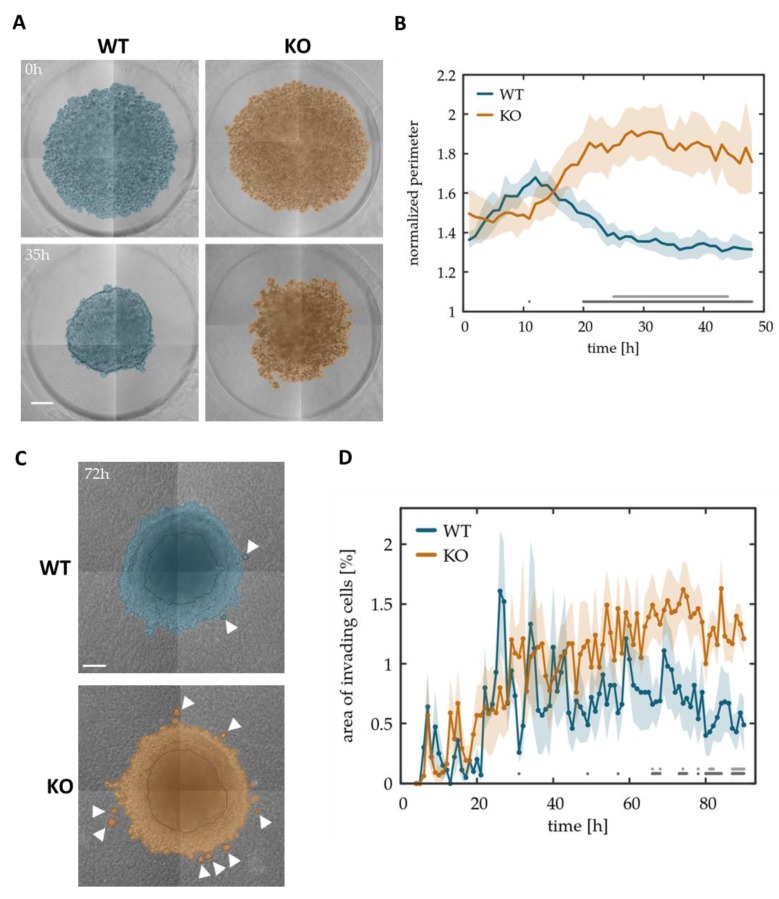
Tks4-KO cells form looser aggregates and invade in small clusters. (**A**) Representative spheroids of 3000 WT (WT) and Tks4-KO (KO) cells in non-adhesive agarose wells. Experiment was conducted in triplicates, scale bar represents 100 µm. Color indicates the cell-covered area, identified by our image processing tools. (**B**) Spheroid shape is characterized by the normalized perimeter, the ratio of the actual perimeter and that of a circle with the same area. Solid lines indicate an average of 9 spheroids, the shaded areas represent the SEM. Statistically significant differences between wild type and Tks4-KO cells are indicated at the bottom of the chart. Dark and light gray dots represent a *p*-value lower than 0.05 and 0.01, respectively, obtained by unpaired Student’s t-tests performed at each time point. (**C**) Representative images of spheroids embedded in type-I collagen matrix. Initial spheroid size is indicated with dark outline. Cells leaving the spheroids and invading into the matrix either as solitary cells or in small groups are marked by white arrowheads. (**D**) Time dependence of the area occupied by invading cell clusters, not connected to the central aggregate. Area values were normalized by the total cell-covered area in the micrograph. Solid lines indicate an average of *n* = 3 and *n* = 6 microscopic fields of wild type and Tks4-KO, respectively. The shaded areas represent the SEM. Dark and light gray dots indicate *p*-values lower than 0.05 and 0.01, respectively, established by unpaired Student’s t-tests performed at each time point.

We observed that Tks4-KO spheroids were less compact than the corresponding spheroids made from WT cells. The aggregates are loose at seeding and gradually become more compact indicated by the reduced overall size at 35 h post-seeding. Tks4-KO cells form an irregular perimeter with loosely attached cell clusters (Figure 4A). To characterize spheroid compactness, we developed image analysis tools to extract the shape of spheroids from micrographs. Each spheroid was characterized by the value of its normalized perimeter: the ratio of the actual perimeter and the perimeter of a circle with the same area. This quantitative measure was extracted from images recorded during the aggregation process (Figure 4B and Figure S5C) as well as from 32 distinct aggregates (Figure S8). The results indicate that wild-type spheroids reach a 50% more compact shape (normalized perimeter = 1.31 ± 0.02) than Tks4-KO cells do (normalized perimeter = 1.76 ± 0.16). The fact that Tks4-KO cells remain in a loose aggregate further supports that cell-cell connections are weak and less functional in the absence of Tks4.

To assess the role of Tks4 in tumor cell invasion, spheroids were embedded into a collagen I matrix and cultures were recorded by automated microscopy. While both WT and Tks4-KO spheroids expanded as cells invaded the collagen I gel, the Tks4-KO cultures exhibited more solitary cells and small cell clusters within the collagen gel (Figure 4C, Video S1). To quantitatively characterize this finding, we segmented the images and identified cell clusters that were not connected to the large central aggregate. During the experiment, the area associated with such solitary clusters increased gradually and reached a value two-fold higher in Tks4-KO cultures than in WT cultures (Figure 4D and Figure S5D). Loss of Tks4, therefore, also correlates with a more solitary invasive phenotype in HCT116 cells.

### 3.5. Reintroducing Tks4 Rescues Altered FN1 and EMT Transcription Factor Expression Levels

**Figure 5:**
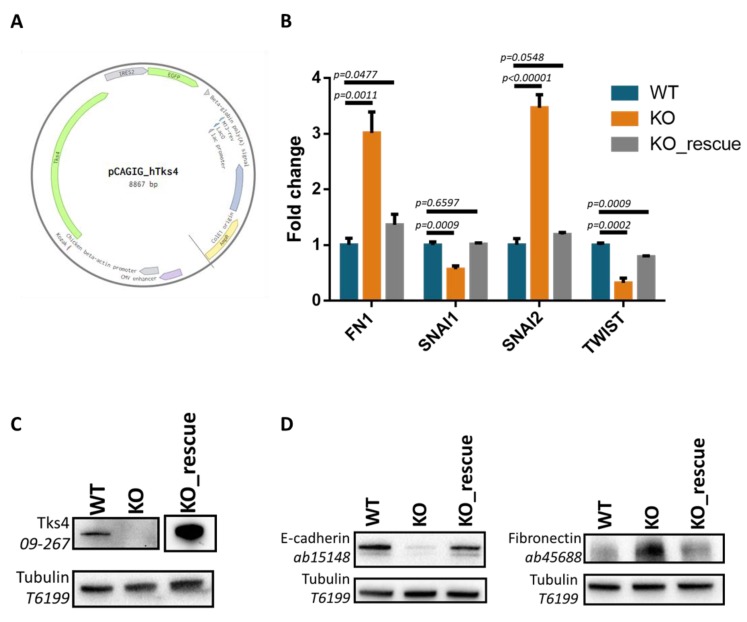
Transfection of Tks4-KO cells with a Tks4 encoding plasmid. (**A**) Tks4 KO cells were transiently transfected with a Tks4 “rescue” expression plasmid. 24 h later, cells were harvested and subjected to qPCR analyses. The expression levels of Snail1 (SNAI1), Snail2 (SNAI2), Twist (TWIST), and fibronectin (FN1) were measured as described in the Materials and Methods. The error bars show the standard deviation, and the statistical analyses were carried out using the unpaired Student’s t-test. (**B**) Schematic diagram of the Tks4-encoding pCAGIG vector. (**C**) Western blot experiments using WT, KO and KO_rescue cell lysates with Tks4 antibody (**D**) Western blot experiments using WT, KO and KO_rescue cell lysates with E-cadherin and Fibronectin antibodies.

To prove that the EMT-like phenotype seen in the KO cells was specifically due to lack of Tks4 rather than to stress responses or other off-target effects of the CRISPR/Cas9 system, HCT116 KO cells were transfected with a Tks4 “rescue” expression plasmid (Figure 5A). Figure 5B shows that the re-expression of Tks4 in the KO cells reverted the altered fibronectin (FN1), Snail1 (SNAI1), Snail2 (SNAI2) and Twist (TWIST) expression levels. To validate the success of our rescue experiment, we checked the Tks4 levels in our KO_rescue cells via Western blotting. The results clearly show the presence of the Tks4 protein in abundant levels (Figure 5C and Figure S9). The altered E-cadherin and fibronectin expression levels were also reverted to the wild type levels following transfection with the Tks4-encoding plasmid (Figure 5D and Figure S10). Since these reversions are coupled to the main mediators and hallmark processes of the EMT program, we concluded that the observed changes were linked to the absence of the Tks4 scaffold protein.

## 4. Discussion

A contribution of Tks4 to cancer progression has been suggested in several cancer types, including breast cancer [53], melanoma [27], acute myeloid leukemia [54], and prostate cancer [55]. Consistent with these links, a detailed analysis showed that Tks4 and its phosphorylation are implicated in invadopodia formation in human colon cancer cells [56]. However, limited information is known about how Tks4 is involved in cancer formation, especially in the epithelial context. Therefore, to investigate the possible functions of Tks4 in detail, human HCT116 colon cancer cells were chosen as a model system since they show functional invadopodia, indicating that they endogenously express Tks4 (Figure 1B) [28,57]. They also express the Tks4-activating Src kinase [58] and can undergo partial EMT [59].

Here we used the CRISPR/Cas9 system to knock Tks4 out and investigate the morphologic, phenotypic and transcriptional changes which developed in the lack of the protein. Our results showed that loss of Tks4 initiated EMT or EMT-like phenotypic and molecular changes in this human colorectal carcinoma cell line. The mechanism by which the loss of Tks4 modulates this EMT-like process in tumor cells remains obscure. One possible explanation is that Tks4 is involved in the regulation of cancer cell motility, as indicated by its roles in EGFR signaling [24,60] an EMT-inducing molecular pathway. It has been already shown that the induction of the EGF/EGFR axis can initiate EMT in cholangiocarcinoma (CCA) cell lines and in the human embryonal rhabdomyosarcoma cell line [61]. In these experiments, increases in the Snail2/Slug and ZEB1 levels were observed upon EGF stimulation. Another group found that EGF upregulates Snail1/2 and ZEB1 expression but does not affect Twist in serous borderline ovarian tumor cells (SBOT) [62]. Both of these publications also mentioned that EGFR induction decreased E-cadherin levels. We assume that the lack of Tks4 likely alters EGFR signaling in these cells, perhaps resulting in a shift toward a mesenchymal-like motile phenotype. Moreover, due to the loss of Tks4 having similar effects on cancer cells as EGF treatment (EMT induction, reduced E-cadherin expression), it is possible that Tks4 plays a putative negative regulatory role in the EGFR pathway by delaying signal transduction or changing its kinetics.

Another possible mechanism by which Tks4 could participate in these EMT-like changes relies on the fact that Tks4 is part of a reactive oxygen species (ROS)-producing protein complex. An early observation from Gianni et al. showed that NoxA1 (NADPH oxidase activator subunit) and Tks4 can interact and that their phosphorylation status influences ROS production [56]. Since ROS is an important player in EMT in colon cancer cells [63,64], we propose that if the signaling pathway that converges on the Tks4 scaffold protein is disturbed, the balanced ROS level is also shifted leading to EMT-associated mechanisms.

A possible role of the Tks scaffold protein family in the formation of invadopodium and metastasis was proposed recently [27]. Invadopodia are actin-rich membrane protrusions, which are instruments of cellular invasion via ECM degradation. As described previously, the loss of Tks5 (a close relative of Tks4) completely diminishes invadopodium formation, while the absence of Tks4 negates ECM decomposition. Specifically, Tks4 as a critical invadopodium marker protein has been also connected to cancer and was used to monitor the invasiveness of colon and breast cancer cells both in vitro and in vivo [47,50]. These seemingly controversial functions of Tks4 could be explained by the theoretical multi-purpose nature of the protein. It is possible that temporary loss of Tks4 has a significant effect on cell migration, which heavily depends on podosome formation, while the permanent absence of the protein in epithelial cells increases their invasiveness by activating an EMT-like program. We hypothesize that the loss of Tks4 has different effects in epithelial and mesenchymal cells. Previous studies used mesenchymal or non-epithelial cell lines to study the effects of Tks4 knockout [23,27,65], and stable and complete knockout of Tks4 in epithelial cells has never been achieved or published before according to our knowledge. We are not challenging the role of Tks4 in podosome/invadosome formation. Instead, we are reporting new and important, but also different, functions for this protein in the regulation of cell morphology and behavior. Based on the available data related to the roles of Tks4 in development, disease, and cancer cell behavior, it appears that Tks4 is important for podosome formation and invasion; however, stable and complete absence of the protein in epithelial cells initiates EMT-like changes.

Despite the concordant results included in the current article, there are several limitations that have to be reckoned with in our study. The experiments were performed on only one epithelial cell line (HCT116), however, the same results were obtained from several clones after Tks4 knock out. The selected cell line has a known deficiency in the epithelial-to-mesenchymal transition process, which makes it an imperfect candidate to study full-blown EMT [59]. Unfortunately, we were not aware of the surprising effect of the absence of Tks4 on EMT at the time we established the cell line. We did not conduct experiments where the EGF or any other possibly affected pathways were disrupted to reveal a plausible mechanistic link between the loss of Tks4 and EMT. Finally, the current study lacks animal experiments to prove the increased EMT-mediated *in vivo* invasiveness of the newly established cell line.

Our results suggest a novel role for Tks4. Given the fact that Tks4 is a scaffold protein without known enzyme activity, the significant changes caused by its loss of function are quite surprising. Knowing how important EGFR signaling and mutations are in physiological and pathological processes, we can presume that a protein involved in this pathway has a critical role to play also. Mutations of Tks4 are associated with the rare but severe Frank-ter Haar syndrome. Patients affected by this congenital disease always show developmental abnormalities, therefore the involvement of an altered EGFR pathway could be also suspected. Though the mechanism which interconnects loss of Tks4 and EMT induction has yet to be understood, our results clearly show a correlation between the two. We propose that Tks4 and its downstream signaling partners are important modulators of the EMT process at the gene expression level. Our hypothesis that Tks4 is a novel molecular regulator of EMT via its roles in altering the expression levels of EMT master regulators warrants further examination.

As our results suggest, the absence of Tks4 in epithelial cancer cells can lead to EMT-like features, increased motility and spreading; therefore, targeting the protein or stabilizing its expression levels could be beneficial strategies for future anti-cancer therapies.

## Supplementary Materials

The following are available online at https://www.mdpi.com/2073-4409/8/11/1343/s1, Figure S1: Genotyping with Eco72I, Figure S2: CRISPR/Cas9 modified alleles of SH3PXD2B gene pasted into the wild type open reading frame Figure S3: Original uncropped western blot pictures of membranes displayed in Figure 1, Figure S4: Original uncropped western blot pictures of membranes displayed in Figure 2, Figure S5: *P*-values of cell motility measurements and spheroid analysis experiments, Figure S6: Average confluency of WT and Tks4-KO HCT116 cultures, as a function of time, Figure S7: Time development of cluster size distribution in Tks4-WT and Tks4-KO monolayer cultures, Figure S8: Number of invading cells from the central aggregate at 3.5 days, Figure S9: Original uncropped western blot pictures of membranes displayed in Figure 5C, Figure S10: Original uncropped western blot pictures of membranes displayed in Figure 5D, Video S1: Time-lapse phase-contrast image sequence of collagen invasion assays performed with WT and Tks4-KO spheroids.
