# Supplementary material for: Absence of the Tks4 Scaffold Protein Induces Epithelial-Mesenchymal Transition-Like Changes in Human Colon Cancer Cells
Source: Cells. 2019 Oct 29;8(11):1343. doi: 10.3390/cells8111343 (PMC6912613; doi:10.3390/cells8111343)
Supplement: Supplementary file 1 [file cells-08-01343-s001.pdf]

cells

Supplementray Material: Loss of the Tks4 scaffold protein induces epithelial-mesenchymal transition-like changes in human colon cancer cells

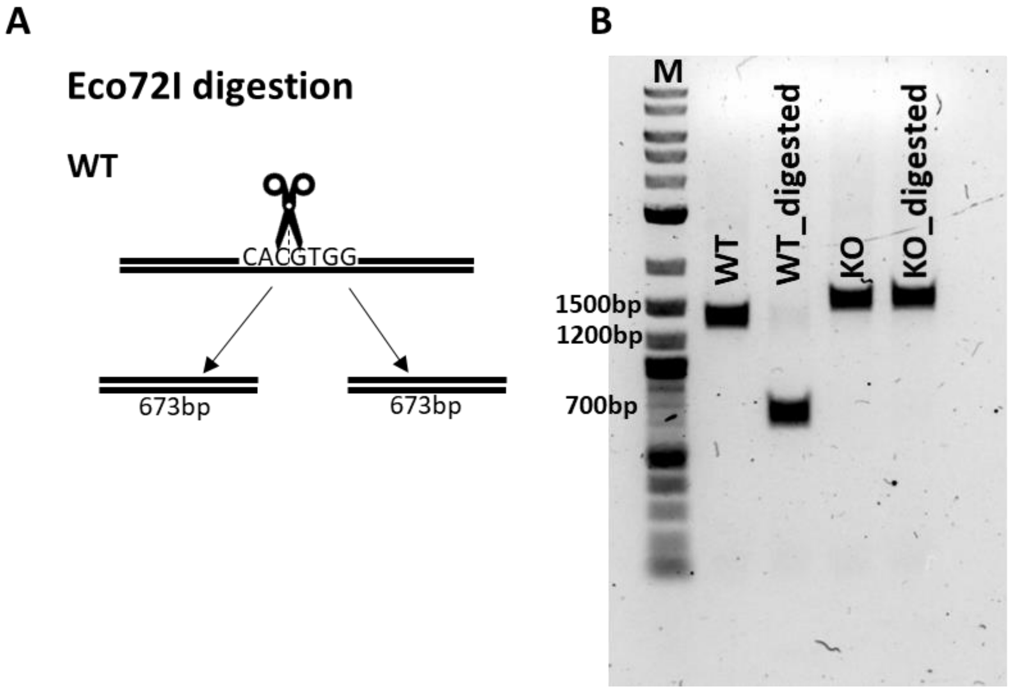

Figure S1. Genotyping with Eco72I. (**A**) Schematic representation of Eco72I digestion in wild-type alleles. Upon digestion PCR products with unmodified exon2 sequences are halved into two 673 basepair long fractions. (**B**) Comparsion of WT and KO, undigested and digested sequences. Wild type sequences are effectively halved, while KO sequences remainintact. M= Quick-Load® Purple 1 kb Plus DNA Ladder (New England Biolabs).

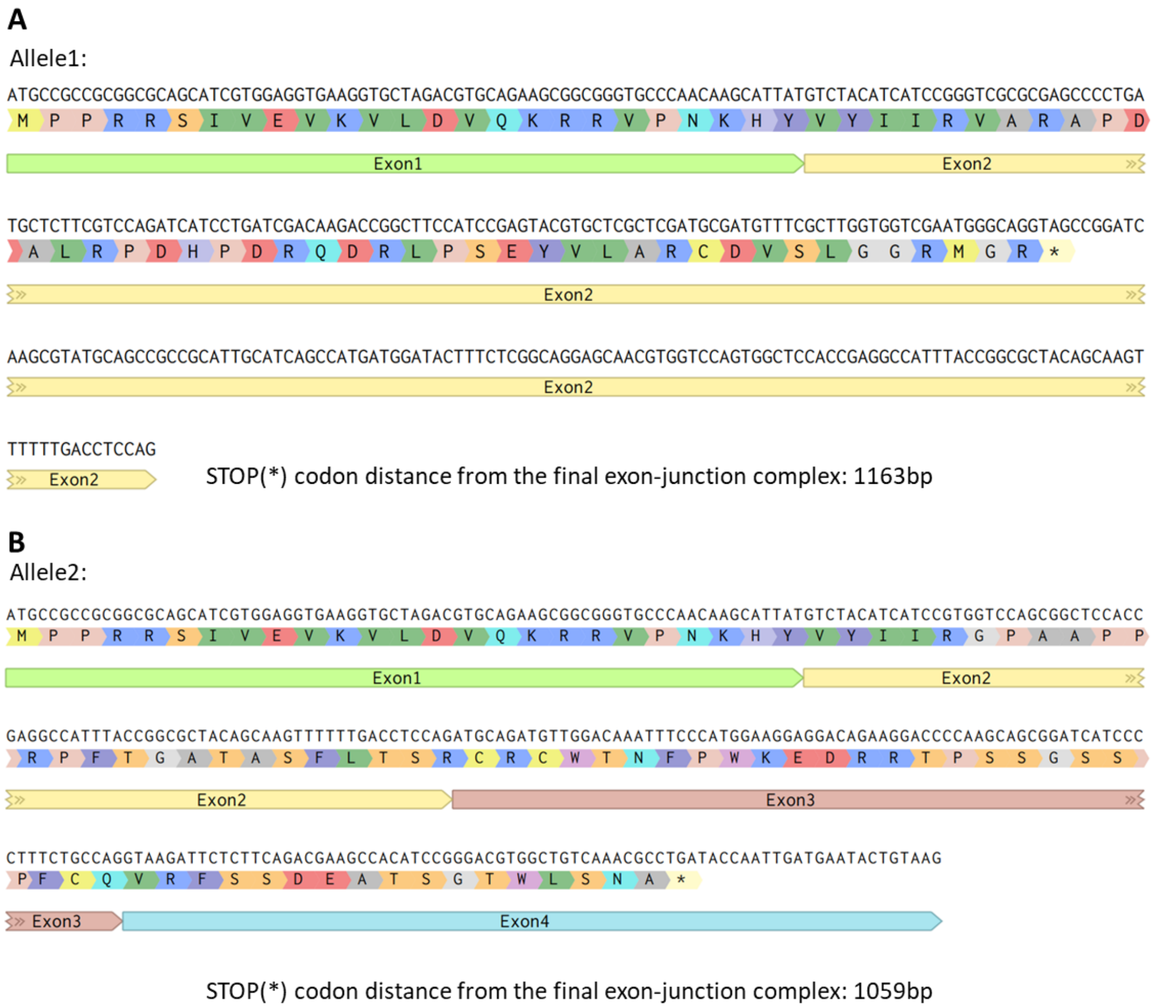

Figure S2. CRISPR/Cas9 modified alleles of *SH3PXD2B* gene pasted into the wild type open reading frame. (**A**) The modified second exon in Allele1 causes a premature STOP codon 1163 bases upstream from the final exon-junction complex. (**B**) The modified second exon in Allele2 causes a premature STOP codon 1059 bases upstream from the final exon-junction complex.

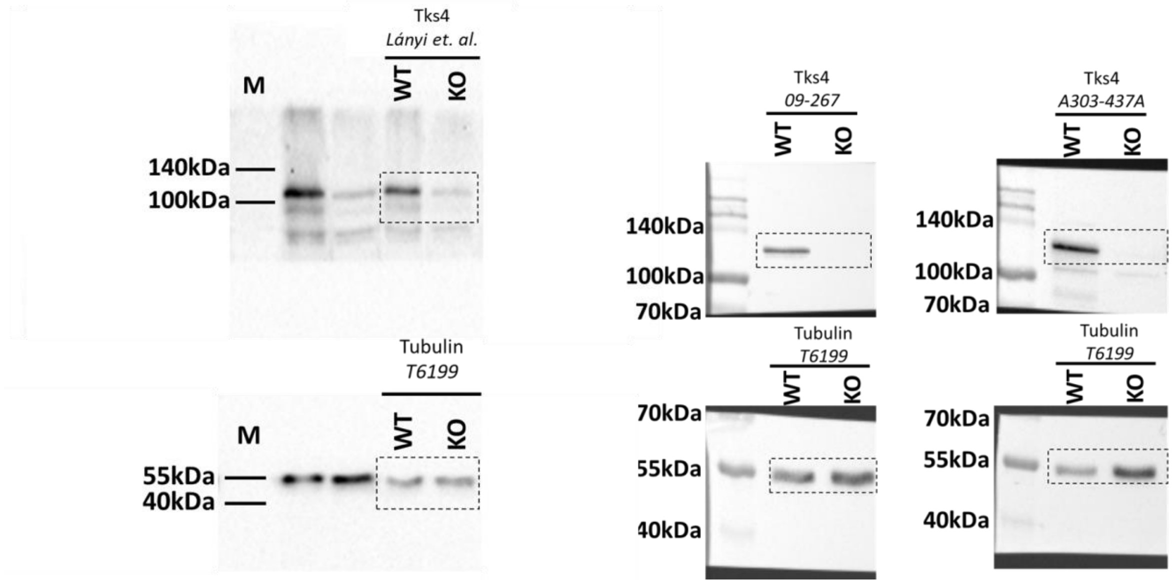

Figure S3. Original uncropped western blot pictures of membranes displayed in Figure 1

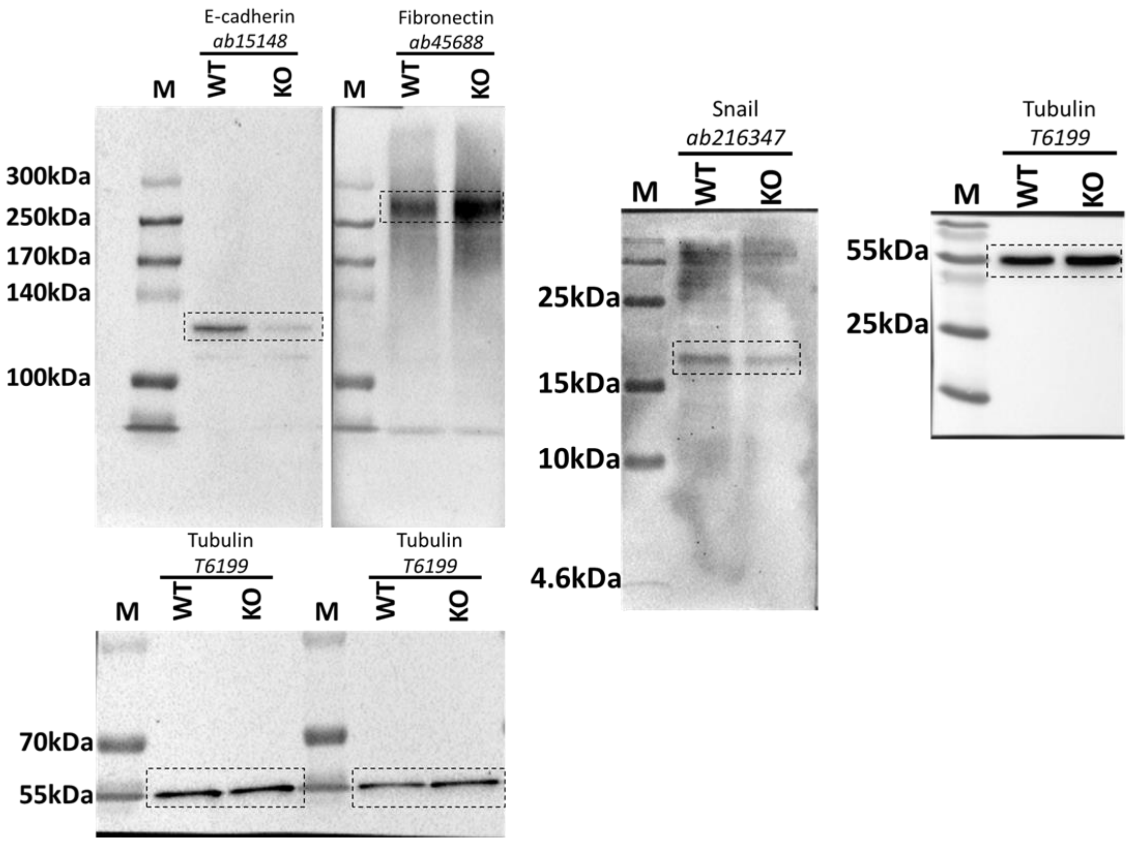

Figure S4. Original uncropped western blot pictures of membranes displayed in Figure 2.

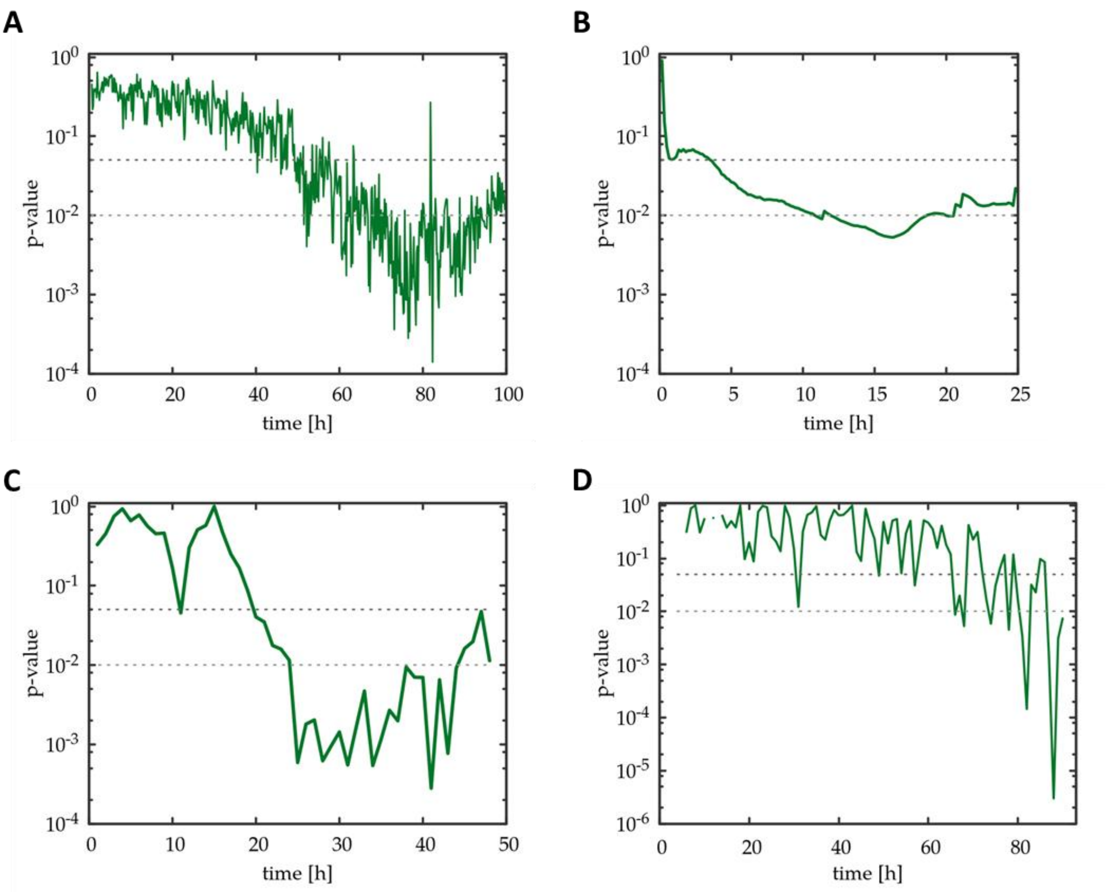

Figure S5. P-values of cell motility measurements and spheroid analysis experiments. (**A**) P-values of experimental data displayed in Figure 3A. Significance (p-value) of difference between the average speeds of wild type and Tks4-KO cells, calculated for each time point of two dimensional random motility assays. P-values were obtained using unpaired Student's t-tests, calculated for a pool of n=27 microscopic fields. (**B**) P-values of experimental data displayed in Figure 3B. Significance (p-value) of difference between the average net cell displacements of wild type and Tks4-KO cells, calculated for each time point of two dimensional random motility assays. P-values were obtained using unpaired Student's t-tests, calculated for a pool of n=20 manually followed cells. (**C**) P-values of experimental data displayed in Figure 4B. Significance (p-value) of difference between the normalized perimeter of wild type and Tks4-KO cells, calculated for each time point of spheroid formation assays. P-values were obtained using unpaired Student's t-tests, calculated for a pool of n=9 microscopic fields. (**D**) P-values of experimental data displayed in Figure 4D Significance (p-value) of difference between the average speeds of wild type and Tks4-KO cells, calculated for each time point of three dimensional collagen invasion assays. P-values were obtained using unpaired Student's t-tests, calculated for a pool of n=3 wild type and n=6 Tks4-KO microscopic fields.

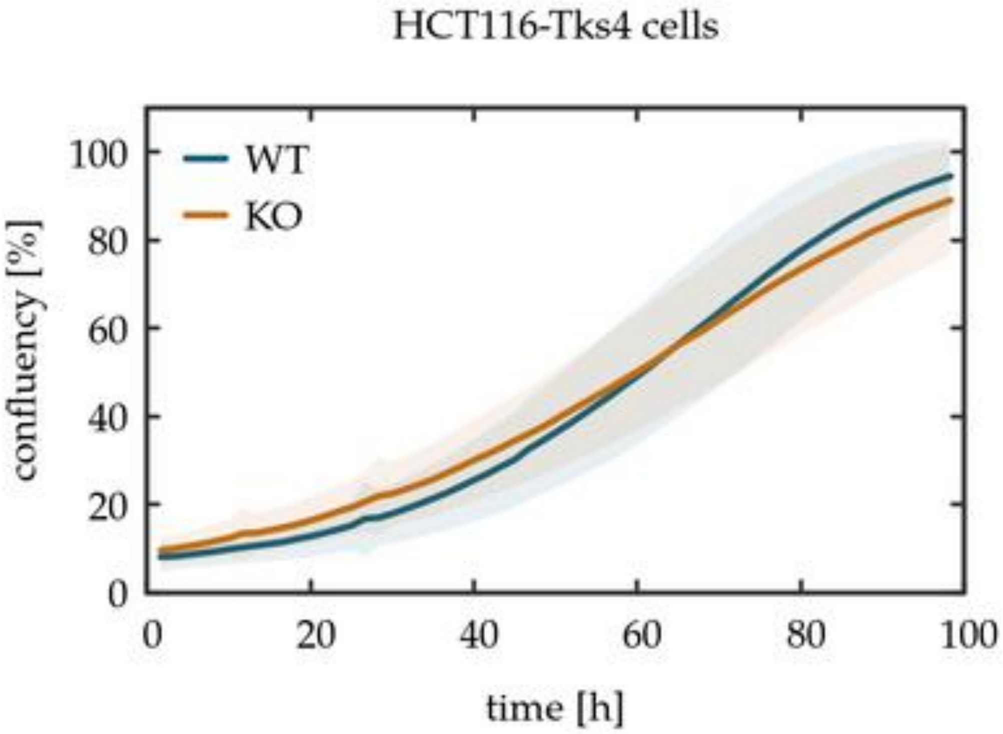

Figure S6. Average confluency of Tks4-WT and Tks4-KO HCT116 cultures, as a function of time. Solid lines represents an average of n=3 independent measurements, each containing 27 microscopic fields. shaded area represents SEM.

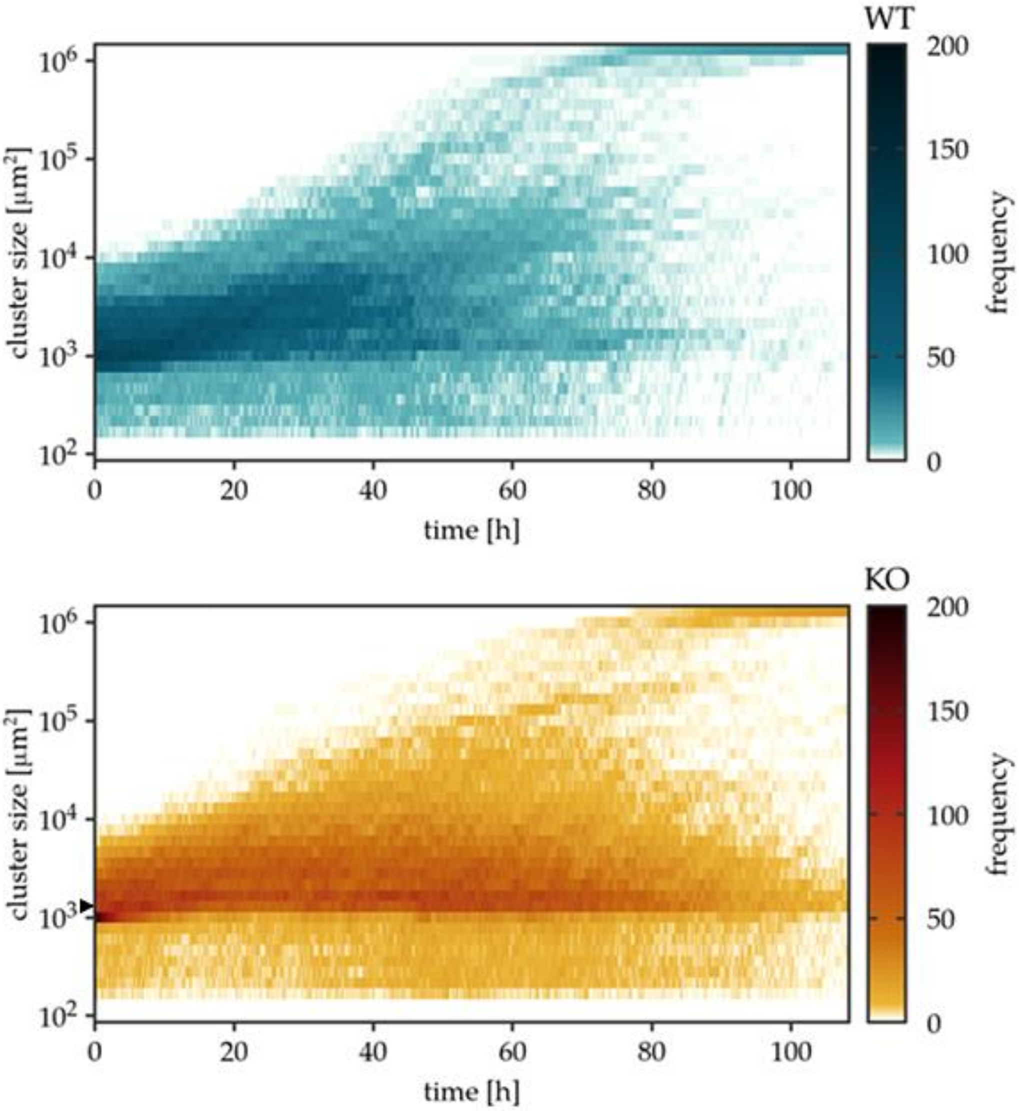

Figure S7. Time development of cluster size distribution in Tks4-WT and Tks4-KO monolayer cultures. The shade of color indicates cluster counts according to the calibration palette. Single cells and small cell clusters are prevalent in Tks4-KO cultures, as reflected by the persistent peak of the distribution at 1000 μm² (arrowhead).

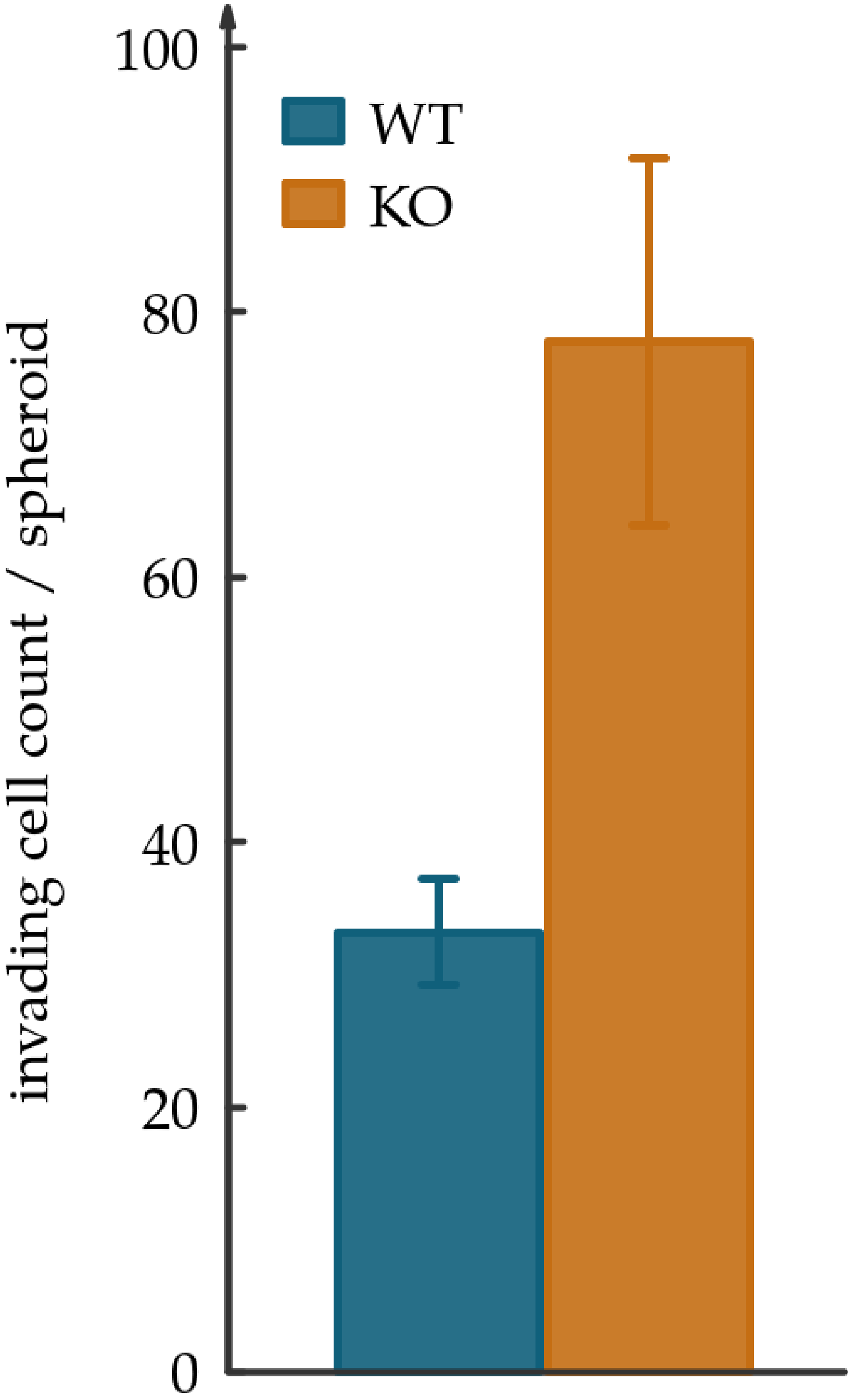

Figure S8. Number of invading cells from the central aggregate at 3.5 days. Error bars represent SEM, sample size is n=18 and n=14, for wild type and Tks4-KO spheroids respectively. Statistical analysis yielded p<0.0036, by using unpaired Student's t-test.

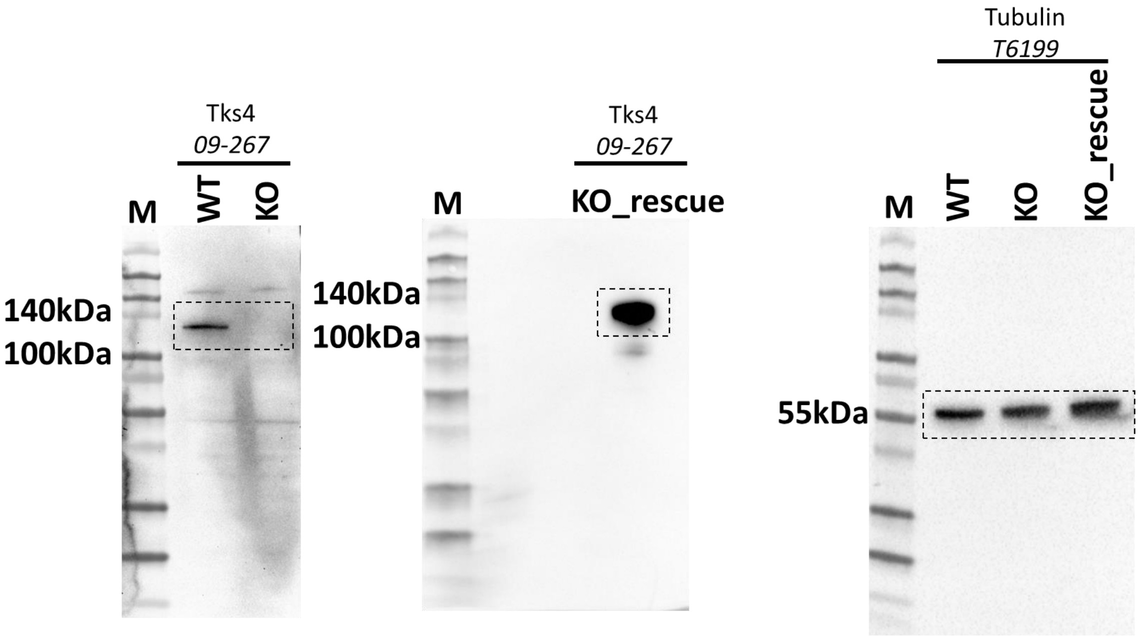

Figure S9. Original uncropped western blot pictures of membranes displayed in Figure 5C.

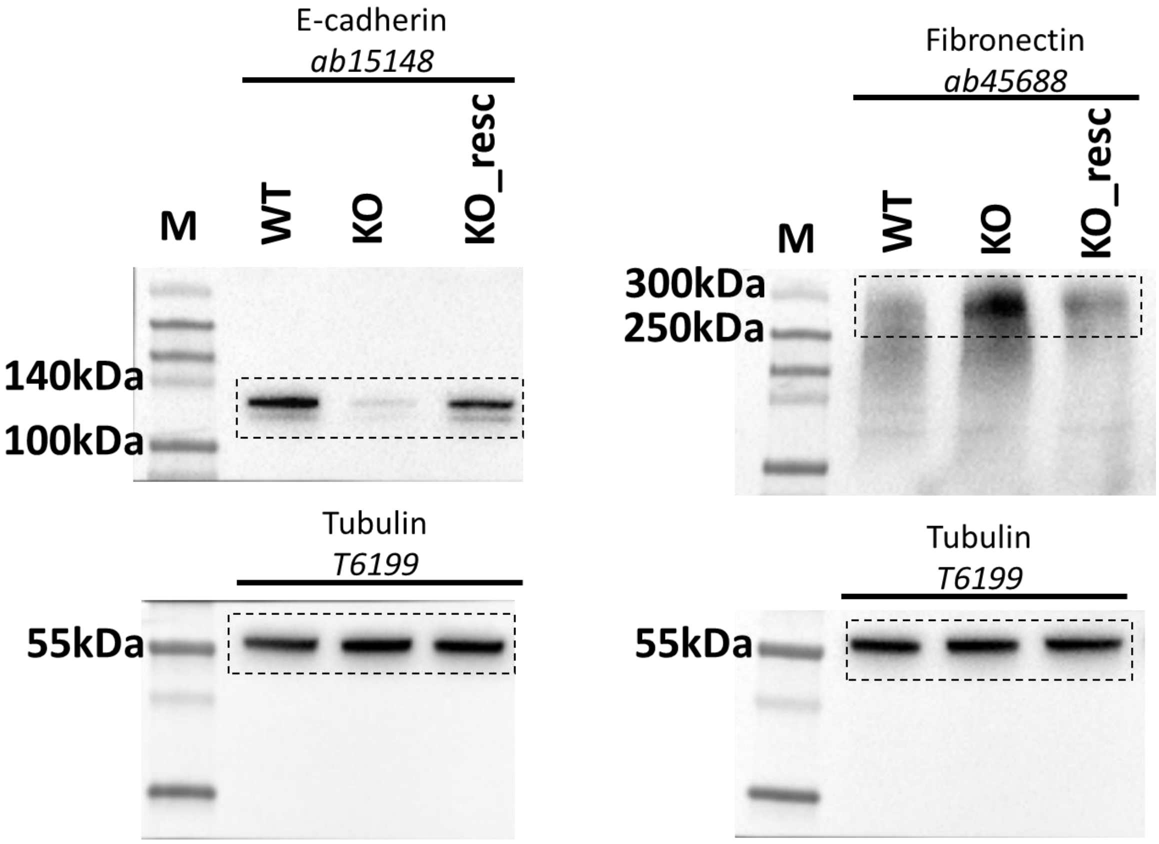

Figure S10. Original uncropped western blot pictures of membranes displayed in Figure 5D.
